# Cold-Water Coral Distributions in the Drake Passage Area from Towed Camera Observations – Initial Interpretations

**DOI:** 10.1371/journal.pone.0016153

**Published:** 2011-01-25

**Authors:** Rhian G. Waller, Kathryn M. Scanlon, Laura F. Robinson

**Affiliations:** 1 School of Ocean and Earth Sciences and Technology, University of Hawaii at Manoa, Honolulu, Hawaii, United States of America; 2 United States Geological Survey, Woods Hole, Massachusetts, United States of America; 3 Marine Chemistry and Geochemistry Department, Woods Hole Oceanographic Institution, Woods Hole, Massachusetts, United States of America; University of Aberdeen, United Kingdom

## Abstract

Seamounts are unique deep-sea features that create habitats thought to have high levels of endemic fauna, productive fisheries and benthic communities vulnerable to anthropogenic impacts. Many seamounts are isolated features, occurring in the high seas, where access is limited and thus biological data scarce. There are numerous seamounts within the Drake Passage (Southern Ocean), yet high winds, frequent storms and strong currents make seafloor sampling particularly difficult. As a result, few attempts to collect biological data have been made, leading to a paucity of information on benthic habitats or fauna in this area, particularly those on primarily hard-bottom seamounts and ridges. During a research cruise in 2008 six locations were examined (two on the Antarctic margin, one on the Shackleton Fracture Zone, and three on seamounts within the Drake Passage), using a towed camera with onboard instruments to measure conductivity, temperature, depth and turbidity. Dominant fauna and bottom type were categorized from 200 randomized photos from each location. Cold-water corals were present in high numbers in habitats both on the Antarctic margin and on the current swept seamounts of the Drake Passage, though the diversity of orders varied. Though the Scleractinia (hard corals) were abundant on the sedimented margin, they were poorly represented in the primarily hard-bottom areas of the central Drake Passage. The two seamount sites and the Shackleton Fracture Zone showed high numbers of stylasterid (lace) and alcyonacean (soft) corals, as well as large numbers of sponges. Though data are preliminary, the geological and environmental variability (particularly in temperature) between sample sites may be influencing cold-water coral biogeography in this region. Each area observed also showed little similarity in faunal diversity with other sites examined for this study within all phyla counted. This manuscript highlights how little is understood of these isolated features, particularly in Polar regions.

## Introduction

Since the opening of the Drake Passage, some 30 million years ago [1], [2], [3] the Antarctic Circumpolar Current (ACC) is thought to create a biogeographical barrier to species dispersal, limiting the transport of benthic larvae between the South American and Antarctic continental shelves. The Drake Passage in particular is a highly dynamic environment, where fast currents are likely to hinder meridional larval transport [4]. Antarctic benthic fauna have been thought to be “cut off” from South American fauna since this time, and therefore have a long history of *in situ* evolution on the deep continental shelf [5], [6]. Despite this separation, faunal similarities have been found in both fossil and live benthic groups bringing into question these theories of larval isolation [5], [7]. However, to date little is known about the benthic fauna that lives in between these two shelves, within the Drake Passage itself. Though the majority of the passage lies in abyssal depths of over 3000 m, there are numerous ridges and seamounts (from ETOPO 2 bathymetry), largely unexplored lying at depths similar to the two adjacent continental shelves (500–1500 m).

The enhanced productivity around seamounts and other geologic features that rise from bathyal and abyssal depths creates hotspots of diversity for both benthic and pelagic organisms [8]. Seamounts in particular can support a fauna that is distinct from that of the surrounding seafloor, potentially supporting both high levels of endemism and recruits from the regional gene pool [8]. The nutrient-rich upwelling, topographic trapping of migrating zooplankton [9] and the influence of strong current regimes also make these areas suitable habitat for filter-feeding cold-water corals [10]. These coral ecosystems are important for increasing local diversity and are used as nursery, feeding and protection areas for potentially thousands of associated species [11]. Despite their importance as ecosystem engineers and recent increased interest in these habitats, the ecology and biogeography of cold-water corals are poorly known, particularly in relation to their shallow-water counterparts. Furthermore, the linkages between environment and habitat, and larval settling and dispersal on remote and hard to sample seamounts is still not understood.

The Drake Passage has over 20 seamounts and ridges (peaks of shallower than 2000 m depth) between the southern extension of the South American shelf and the Western Antarctic Peninsula shelf, but the passage is also notorious for large waves, fierce storms and strong currents, making benthic sampling difficult and therefore infrequent. Seafloor photographs, while not a replacement for physical samples, can help supplement the small number of samples that have been obtained from this challenging environment. This paper presents the first data on *in situ* photographs taken during research cruise NBP08-05 in May 2008, from seamounts and ridges within the Drake Passage.

## Methods

**Figure 1:**
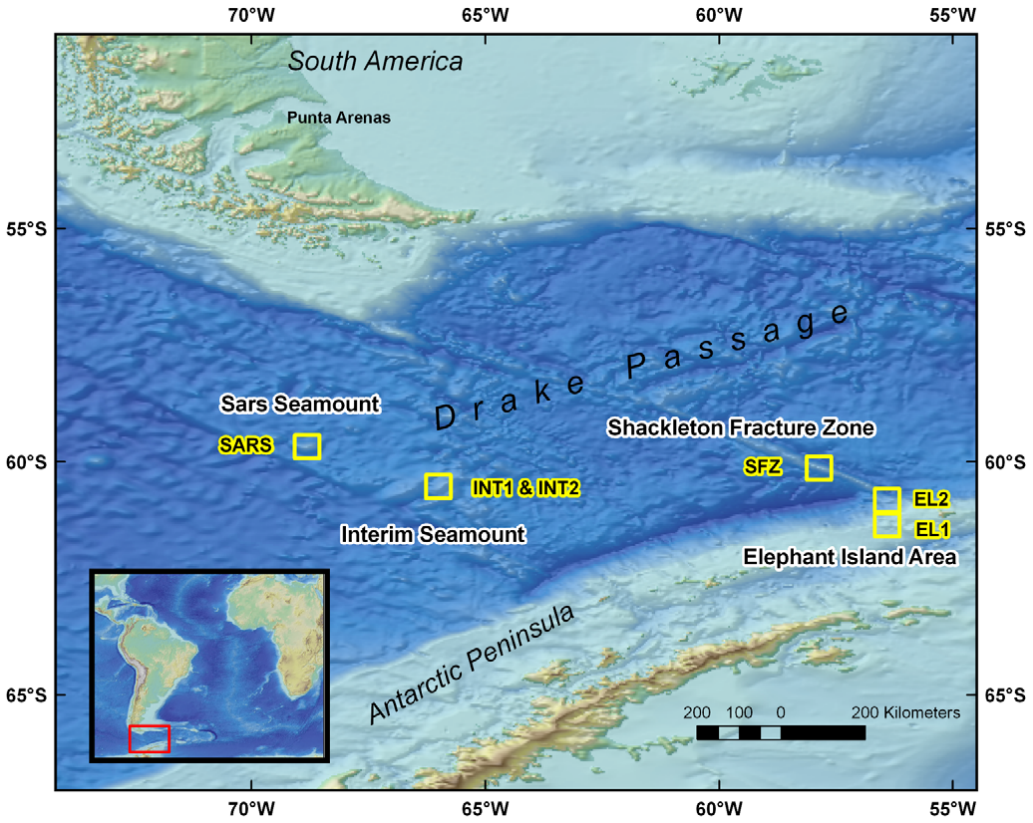
Map of locations in the Drake Passage where towed camera surveys were conducted (yellow boxes).

During cruise NBP08-05 on the *ARV Nathaniel B. Palmer*, the Woods Hole Oceanographic Institution (WHOI) TowCam (Deep Sea Power and Light DigiSeaCam, 11) was used to collect high-resolution digital photographs from six sites in four areas across the Drake Passage (Fig. 1). The onboard SBE-25 CTD (Conductivity, Temperature, Depth Sensor, Seabird Electronics) with altimeter allowed this system to be towed consistently at the optimal distance above bottom (∼5 m) to collect an image area approximately 2.5×1.5 m.

Towed camera surveys were conducted at six locations (Fig. 1, Table 1) – the continental margin off Elephant Island at 450 m (EL-1) and at 1900 m (EL-2); along the Shackleton Fracture Zone at 800 m(SFZ); Interim Seamount at 1175 m (INT-1) and 1500 m (INT-2); and Sars Seamount at 610 m (SARS). The WHOI TowCam took photos every ten seconds (average ship speed 0.25 m/s), allowing for image overlap to mosaic areas of interest. Surveys were run in a straight line from 1.9–4 km long, and took between 1000–1700 digital images per tow (see Table 1.).

**Table 1:** Locations of Towed Camera surveys.

				Start Point		Altimeter		
Name	Location	Length (km)	Depth (m)	Lat (S)	Long (W)	Height (m)	+/−SD	No. Images
EL-1	Elephant Island Area	2.7	400–450	61 17.03	56 25.98	4.7	0.44	1410
EL-2	Elephant Island Area	3.5	1850–1900	60 52.25	56 24.28	5.7	3.67	1667
SFZ	Shackleton Fracture Zone	1.1	700–800	60 10.86	57 50.07	5.09	0.6	1052
INT-1	Interim Seamount	3.5	1030–1175	60 34.61	66 00.19	5.2	0.94	1554
INT-2	Interim Seamount	2.2	1340–1500	60 31.61	65 54.96	5.09	0.74	1058
SARS	Sars Seamount	3.4	490–610	59 42.93	68 43.80	5.06	0.72	1717

**Figure 2:**
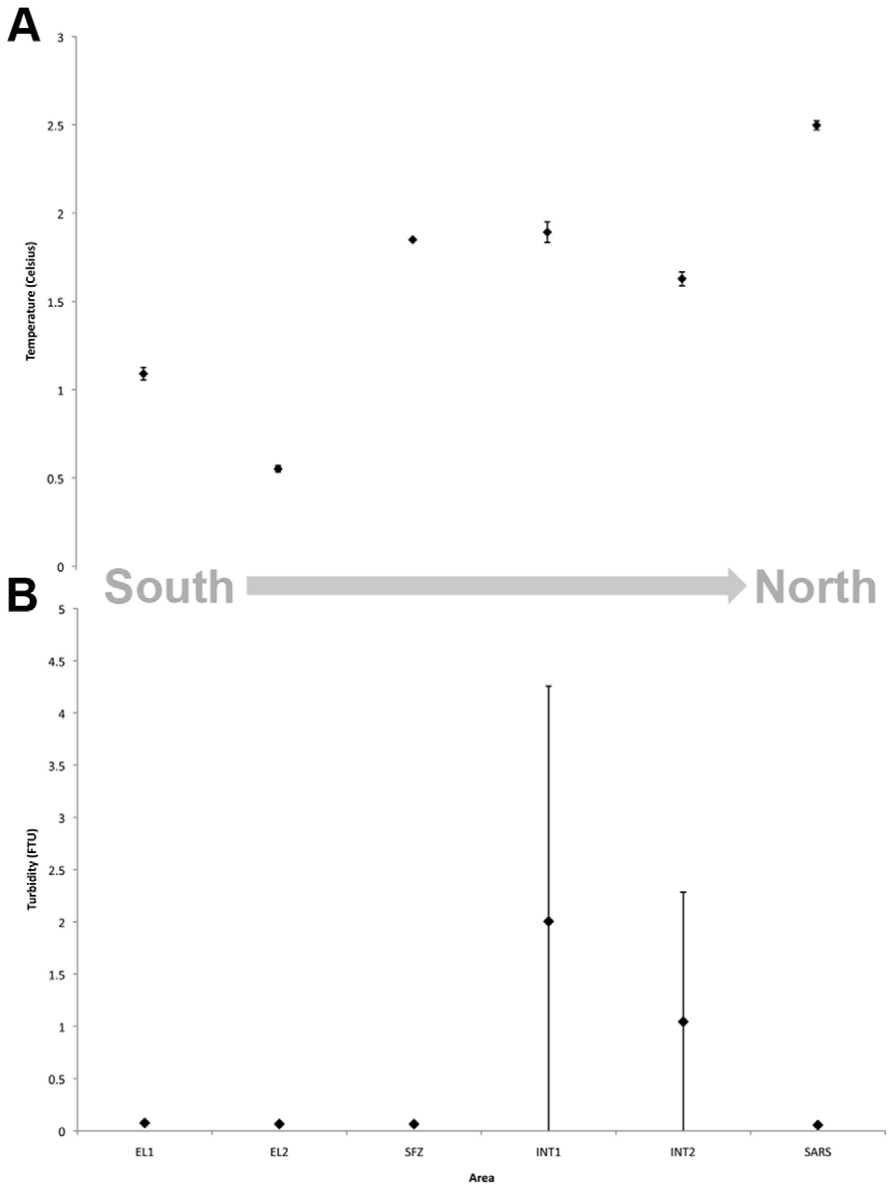
Environmental data acquired throughout each survey by CTD instrumentation on the camera sled. A, Average temperature along tow; B, Average turbidity along tow. Error bars show standard deviations for each tow. The variability in SD for turbidity for Interim Seamount sites shows that rather than a constant high level of particle load, there were pockets of turbidity seen throughout the tow. These high spikes did not correspond to times when the camera sled touched the seafloor, and so do represent an accurate pattern of higher turbidity at these sites.

**Figure 3:**
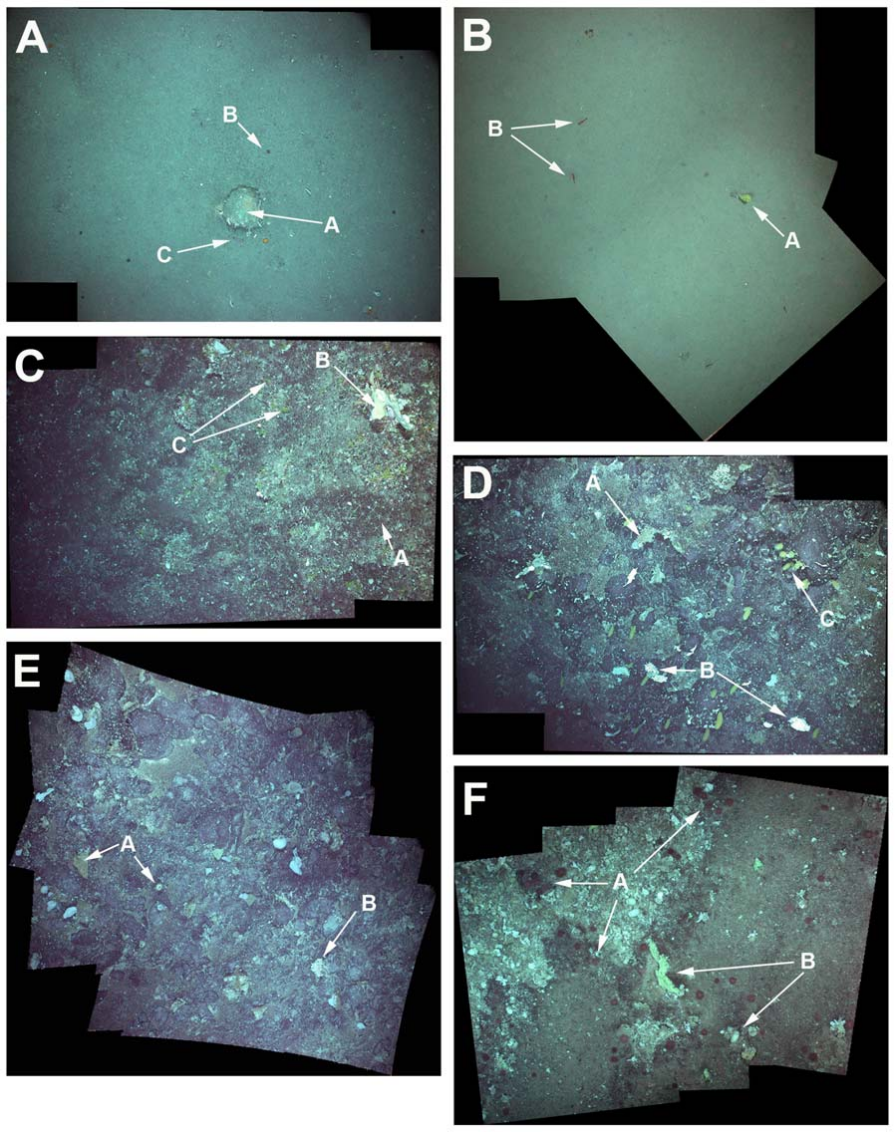
Mosaics of representative images from each of the towed camera surveys. A single TowCam image covers approximately a 2.5×1.5 meter area of seafloor; these mosaicks are made up of 2–4 images. A, EL-1; Image shows the predominantly fine-grained sediment environment of EL1, with occasional drop stones (A) covered in anthozoans (anemones). In the sediment the Alcyonoacean *Anthomastus bathyproctus* can be seen (red circle above rock, B) and numerous brittle stars, as well as a holothurian (purple below rock, C). B, EL-2; Image shows predominately sediment environment of EL2 with the primnoid octocoral *Thouarella* sp. (yellow bottlebrush, A) present on small rock, and numerous red shrimp (B). C, SFZ; Image shows predominately gravel bottom habitat with patches of sediment, dominated by brittle stars (A), large sponges (white fans, B) and primnoa octocorals (yellow bottlebrushes, C). D, INT-1; Image shows gravel bottom habitat with exposed bedrock in places. Bottom fauna is dominated by sponges (white/brown fans, A), stylasterids (bright white fans, B) and primnoa octocorals (yellow bottlebrushes, C). E, INT-2; Image shows primarily bedrock environment with patches of sediment and gravel. Bottom fauna shows large sponges (white/brown fans and tubes, A) and stylasterids (white fans, B). F, SARS; Images shows gravel bottom dominated by anemones (red circles, A) and sponges (yellow/white/brown tubes and fans, B).

For image analysis, all image metadata and corresponding CTD data from each tow were exported to Microsoft Excel and images taken more than 5.5 m, or less than 3.5 m above bottom were discarded. Environmental data were also collected (temperature and turbidity, Seapoint Sensors Inc.) using the onboard SBE-25 CTD system (Seabird Electronics). Environmental data were taken from when the camera reached the seafloor to when it left, and averaged over the entire tow (Fig. 2). Images from each tow were then examined for poor quality images (e.g. excess turbidity from ‘landing’, over exposure etc. making accurate interpretation impossible) and for duplicate images, which were also discarded from analysis. Two hundred of the remaining images selected randomly (using a random number generator in Microsoft Excel) from along the survey line were then examined individually, avoiding any overlapping images (to avoid double counting). Overlapping images were mosaicked using Microsoft Image Composite Editor (ICE, V1.2r1, Fig. 3a–f) for qualitative assessment.

**Figure 4:**
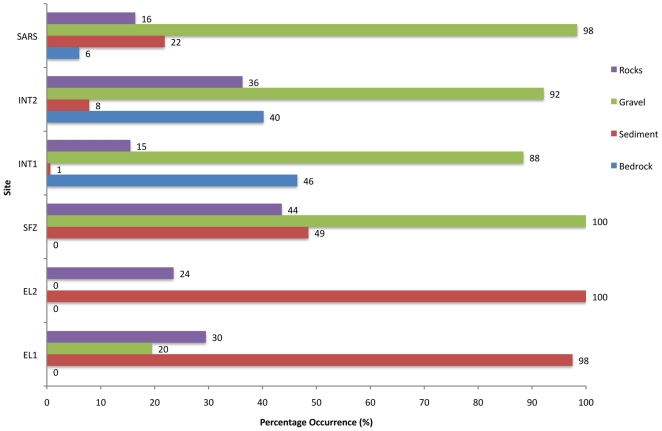
Bar graph representing dominant bottom types for each survey. Results are based on percentage occurrence of bottom type in 200 seafloor images from each location and bar numbers represent actual values. Bars are arranged with most northern site at the top to the most southern at the bottom.

To analyze habitat differences between tows, the presence of four types of substrate was recorded in each image (Fig. 4). Images with multiple substrate types were scored for presence of each bottom type. Sediment samples were not collected; therefore sediment texture was estimated visually from the photos. For the purposes of this paper the four substrate types were defined as follows: **Sediment** – sand, silt, or clay (grains smaller than about 2 mm); **Gravel** – granules, pebbles and cobbles up to about 0.25 m in diameter; **Rock** – boulders more than about 0.25m on one axis; and **Bedrock** – continuous rocky substrate.

**Figure 5:**
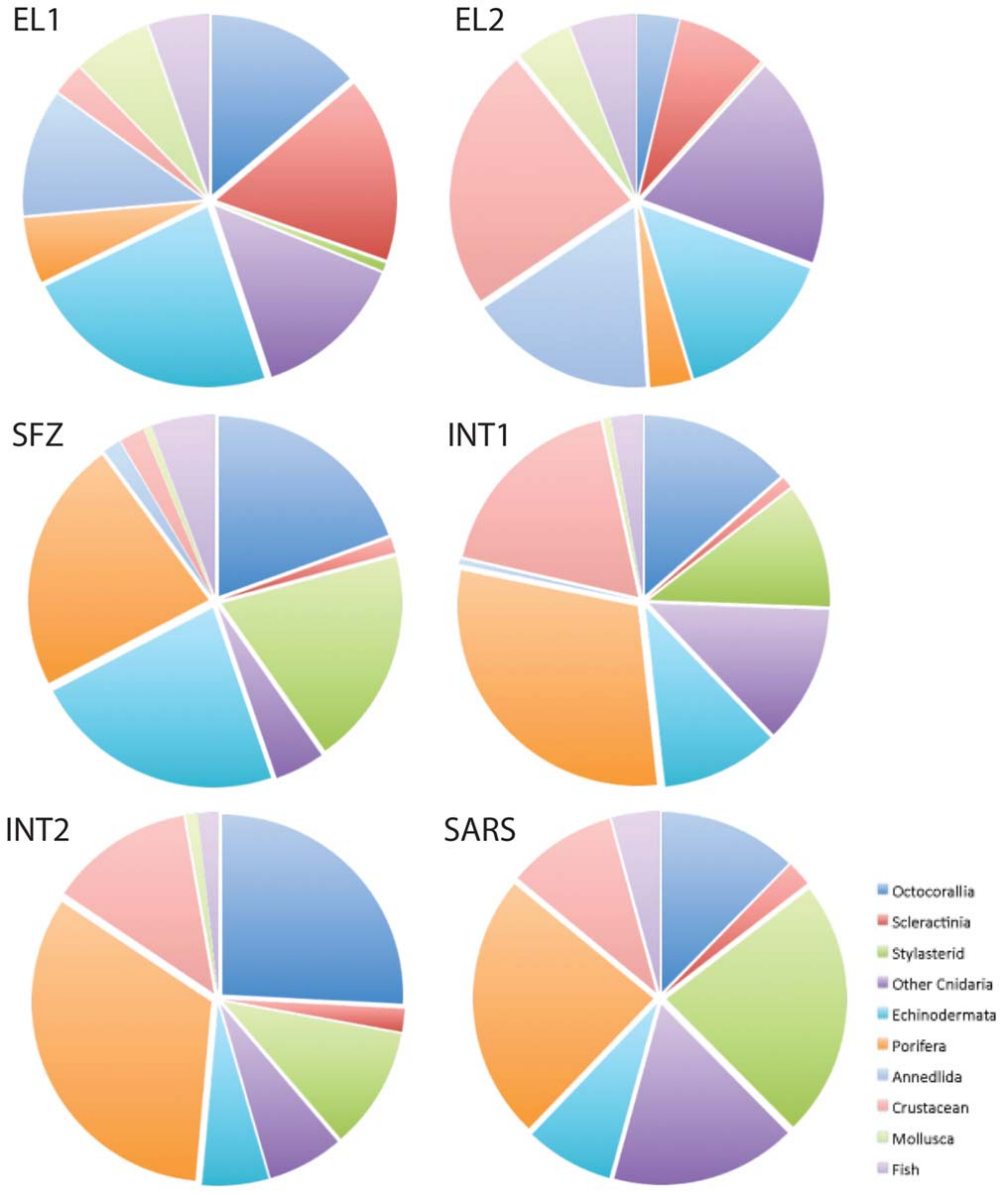
Pie charts show community composition for 6 areas interpreted from 200 randomly selected photos from each area. Images were scored for presence/absence of each taxon, then combined and community composition presented as pie charts.

**Figure 6:**
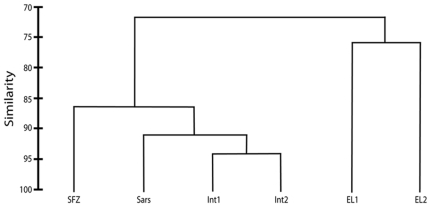
CLUSTER analysis for community composition data, based on Bray Curtis Similarity indices.

**Figure 7:**
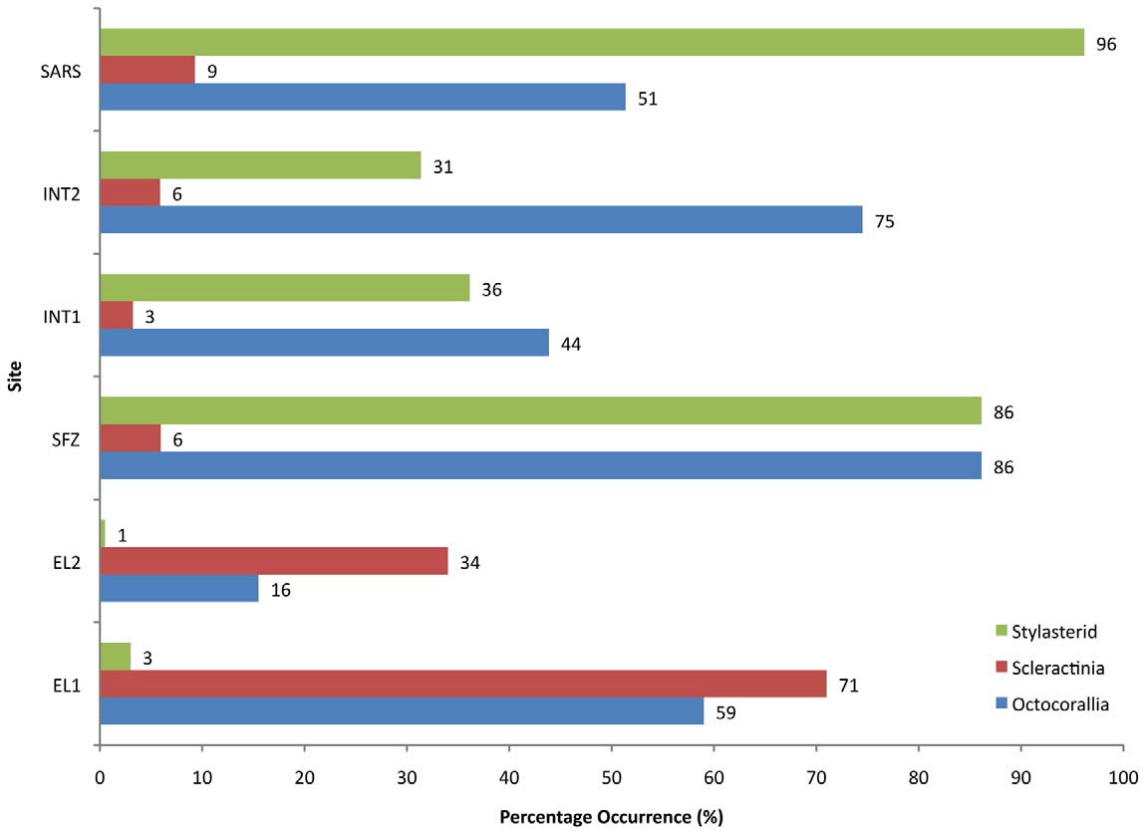
Bar graph showing percentage of coral taxa observed in 200 photos from each tow location (bar numbers represent actual values). Note the relative importance of scleractinia at EL1 and EL2 compared to the importance of stylasterids at the other sites. Bars are arranged with most northern site at the top to the most southern at the bottom.

Presence/absence of the following faunal groups were also recorded from each image – **Octocorallia**, **Scleractinia**, **Stylasteridae**, **Other Cnidaria**, **Echinodermata**, **Crustacea**, **Mollusca**, **Annelida** and **Fish**. Numbers of each group were converted to percentages to show proportions of images each group appeared in, and data combined into pie charts to demonstrate community composition at each location (Fig. 5). PRIMER 6.1.9 (PRIMER-E Ltd.) was used to create a resemblance matrix of Bray Curtis Similarity, using abundance data of phyla (all cnidarian data combined). Data were then used for a CLUSTER analysis using group averages, and presented as a dendrogram (Fig. 6). Coral taxa data were then analyzed separately and presented as percentages per location (Fig. 7). Where possible; corals were identified to species using collections made at sampling locations for qualitative observations; no fauna was identified to that level solely based on towed camera images.

## Results

### Elephant Island -1 (EL-1) – 400 m – 450 m water depth (2.7 km long)

This survey crossed a flat area near the continental shelf-edge west of Elephant Island from the southwest to the northeast. This area is predominantly sediment with occasional rocks (probable glacial drop stones) and gravel areas (Figs. 2, 4a). The average water temperature near the seafloor during the tow was 1°C (+/−SD 0.036) and turbidity was 0.07 Formazin Turbidity Units (FTU) (+/−SD 0.003 - Fig. 2). Cnidarians (71% of images showed scleractinians, 59% of images showed octocorals) were dominant at this location (Figs. 5, 7), though scleractinians were mainly the single species *Flabellum impensum* and the Octocorallia were dominated by the alcyonacean *Anthomastus bathyproctus*. Stylasterids were present in small numbers of images (3%, Fig. 7) primarily attached to rocks present in the sediment. Other dominant fauna were chiefly the echinoderms, with echinoids and ophiuroids present in 98% and 82% of images respectively.

### Elephant Island - 2 (EL-2) – 1850 m – 1900 m water depth (3.5km long)

This tow was also west of Elephant Island in deeper water than EL1. The tow track ran WSW to ENE, parallel to contours on the continental slope, near the southeastern end of the Shackleton Fracture Zone. In this area 100% of the photos had sediment cover with occasional rocks (22% of images, also probable glacial drop stones, Figs. 2, 4b). Average water temperature near the seafloor during the tow was 0.5°C (+/−SD 0.018) and turbidity was 0.06 FTU (+/−SD 0.003 - Fig. 2). This area also showed the lowest total presence of corals of any analyzed sites (Fig. 7). Corals present were primarily *Flabellum spp.* solitary scleractinians (34% of images), the alcyonacean *Anthomastus bathyproctus* (16% of images), with small numbers of stylasterids attached to rocks (1% of images, Fig. 7). Red shrimp, were also present in high numbers at this location (100% of images, Fig. 5). Other cnidarians (actiniarians) were the second largest group in this area present in 80% of images, followed by annelids (71%) and echinoderms (62%).

### Shackleton Fracture Zone (SFZ) – 700 m – 800 m water depth (1.1 km long)

This survey track was in a saddle between two peaks near the southern end of the Shackleton Fracture Zone, at a depth of about 750m. The track was oriented at right angles to the NW – SE trending fracture zone. In this area gravel appeared in 100% of photos, with sediment and rocks also present (Figs. 2, 4c). Average water temperature near the seafloor during the tow was 1.8°C (+/−SD 0.007) and turbidity was 0.06 FTU (+/−SD 0.003 - Fig. 2). Corals at this site were mainly octocorals and stylasterids (both in 86% of images) with scleractinians present in just 6% of images. This site is dominated by sponges and echinoderms (both present in 100% of images).

### Interim Seamount - 1 (INT-1) – 1030 m – 1175 m water depth (3.5km long)

The first survey at Interim Seamount crossed the flanks of three peaks that comprise this elongate seamount. The track ran from north to south and varied in depth from about 1030 m to 1175 m. Gravel dominates the substrate type (88% of images), with exposed bedrock (46% of images) and rocks (15% of images). Very few photos showed sediment during this tow (0.6% of images - Figs. 2, 4d). Average water temperature near the seafloor was 1.9°C (+/−SD 0.059 - Fig. 2). Tows from Interim Seamount showed patches of turbidity (Fig. 2), with an average of 2.004 FTU (+/−SD 2.253). This site showed small proportions of corals, with octocorals (44% of images), chiefly primnoid whip corals, and stylasterids (36% of images) being dominant over scleractinians (3% of images, Fig. 7). Sponges dominated this site (97% of images) with red crabs (59% of images) and other cnidarians (40% of images) making up the other largest faunal groups (Fig. 5).

### Interim Seamount - 2 (INT-2) – 1340 m – 1500 m water depth (2.2km long)

The second survey at Interim Seamount started near the top of the north end of the seamount in about 1340 m and ran downslope toward the west. Gravel (92% of images) and exposed bedrock (40% of images) were most predominant at this deeper Interim Seamount site, with rocks (36% of images) and small patches of sediment (7% of images) also present (Figs. 2, 4e). Average water temperature near the seafloor during the tow was 1.5°C (+/−SD 0.039) and turbidity was 1.043 FTU (+/−SD 1.239 - Fig. 2). Porifera (95% of images) and Octocorallia (75% of images) were present in the highest numbers, with red crabs (crustaceans, 37%) and echinoderms (17% of images) also present (Fig. 5). Primnoid octocorals dominated the coral fauna, with stylasterids (31% of images) and small numbers of solitary scleractinians also present (6% of images, Fig. 7).

### Sars Seamount (SARS) – 490 m – 610 m water depth (3.4km long)

This camera tow started on the upper slope of Sars Seamount and ran across the flat central peak, from south to north. Sars Seamount showed gravel bottom in almost all photos (98.36%) with only small numbers of photos showing sediment (21.8% of images), bedrock (6% of images) or rocks (16% of images, Figs. 2, 4f). Average water temperature near the seafloor during the tow was 2.5°C (+/−SD 0.025) and turbidity was 0.05 FTU (+/−SD 0.004 - Fig. 2). Though octocorals (51% of images) and stylasterids (96% of images) dominated the coral fauna on this seamount (scleractinians were only present in 9% of images), Sars Seamount has a abundant and diverse sponge fauna (99.4% of images, Figs. 5, 7). The other dominant fauna included other cnidarians, such as anemones, which were present in 69% of images and crustaceans present in 41% of images.

### CLUSTER Analysis

CLUSTER analysis (Fig. 6) showed distinct clustering of sites within the Drake Passage (SFZ, Sars, INT1 & 2) separate from the WAP margin sites (EL1 & 2). The two sites on the WAP margin, though more similar to each other than to sites in the Drake Passage, were only 75% similar, likely owing to the large depth difference between these sites (∼1500m). Being geographically closer, as expected, the Shackleton Fracture Zone was most similar to the Western Antarctic Peninsula (WAP) margin sites (72%), though it was still more closely related to Drake Passage locations. Interestingly, the SFZ was more closely related to Sars Seamount than to the geographically closer Interim Seamount, though this could be because of the depth similarity of these two sites.

## Discussion

Though other studies have suggested that seamount communities are most closely affiliated with those on the nearest continental margin [13] this study, even at phylum level, shows a distinct difference in faunal makeup between the Western Antarctic Peninsula (WAP) continental margin sites and sites within the Drake Passage.

Similar to other oceans [14], corals and other cnidarians are found in large numbers on seamounts and ridges in the Drake Passage, as well as on the WAP margin, yet no morpho-species of coral collected during this cruise (many are presently unidentified) were the same between the WAP margin and the Drake Passage (Robinson & Waller, cruise report). It is interesting to note that sponges were present in more than 95% of the images from the four Drake Passage sites, and particularly on Sars Seamount where sponges were present in more photographs than corals. Sponges can colonize habitat suitable for coral growth, and can overgrow coral colonies in shallow-water [15], [16] but it is not known if this occurs in the deep ocean. Deep sponge reefs are important ecosystem builders, attracting similar numbers of associated species as cold-water corals [17]. With the large numbers present in this study, they are likely to be important components of Drake Passage Seamount ecosystems alongside coral communities.

The coral taxa in this study showed potential biogeographic patterns across the Drake Passage in relation to substrate types, and possibly related to the water temperature increase from south to north (0.5–1.0 degrees Celsius at WAP and 1.5–2.5 degrees Celsius at Sars Seamount). Higher turbidity within the Drake Passage may also be a factor, as turbidity and sedimentation in particular can be detrimental to deep-water scleractinians [18]. Though the WAP sites show higher percentages of sedimented habitat, the high currents found within the Drake Passage could be the cause of the higher turbidity at Interim Seamount, lifting and retaining particulates around the topology of the seamount.

Scleractinians are usually found in hard-substrate and high-current-flow environments [11], yet this study found they were the most abundant coral on the sediment-covered WAP margin west of Elephant Island and relatively rare on the rocky seamounts within the Drake Passage. Though all scleractinians do require a hard substrate for larval settlement, this requirement can be satisfied by a small pebble, rock or shell in a predominantly sediment habitat [19]. Of the nine species of scleractinian found on the Antarctic Continental Shelf, seven of these are specially adapted for sedimented habitats [20]. Thus the high abundance of scleractinians on the WAP was expected, and though species data have not yet been compiled from this cruise, the species diversity at the WAP sites does appear to be low, being comprised of primarily *Flabellum spp.* cup corals. Few octocorals and stylasterids are adapted to live in sediment habitats [21], [22] so infrequent observations of those groups on the WAP margin compared to the more current-swept areas of the Drake Passage were also expected. *Anthomastus bathyproctus* is the major exception; this exclusively sediment-dwelling octocoral was found in high numbers (59% of images) at the 400m Elephant Island site. Stylasterids have been reported in high numbers from the Arctic [21], [23] and Patagonia [24] and often dominate deep polar habitats [25], particularly in areas of low nutrients where they are not outcompeted by faster growing organisms [21]. Since there is no published information on the organic nutrient availability at each of these sample locations, it is difficult to determine if this is also a factor in the coral distribution observed in this study. Stylasterids were present in reduced numbers on the WAP margin, likely inhibited by the predominately fine-grained sediments and lack of extensive hard bottom substrates.

Within the Drake Passage, however, hard substrate (in the form of bedrock, gravel and rocks) was more abundant, yet surprisingly the presence of scleractinians was low. It should be noted however, of the seventeen species of scleractinian found in Antarctic waters today, only two form large colonial structures and the remaining fifteen species are smaller solitary-living forms such as species of family Flabellum and Caryophyllia [20]. These smaller scleractinians often occur in cracks and underhangs (pers. obs.), areas that could easily be missed from a downward looking towed imaging system and thus missed from this data set. Attached solitary corals can also often be found in clusters (pers. obs.), and so their influence to an ecosystem is likely to be underestimated in presence/absence data. Future studies can expand this preliminary data set by collecting samples and conducting closer image transects with scaling systems (using drop cameras in tandem with towed packages) to more fully characterize the ecosystem for both biodiversity and abundance data.

The difficulty in sampling habitats in the Drake Passage has led to a paucity of information on benthic fauna, yet on this expedition seamount composition was found to be rich and diverse, particularly with regards to cold-water coral and sponge ecosystems. At each of the sampling locations, corals comprised greater than a quarter of all benthic fauna, demonstrating their importance to these polar communities. None of the species observed were the same between the WAP and Drake Passage sites, suggesting these two areas have two distinct faunal communities. These images of seamounts and ridges in the Drake Passage are the first indication of the diversity of fauna in this region and its potential interdependence on local environmental conditions. Understanding the prime constituents of seamounts and ridges inside the Drake Passage in relation to the South American and Antarctic continental shelves can enhance understanding of larval transport and evolutionary processes in this dynamic area. This is especially important as anthropogenic ocean warming and acidification are threatening these delicate polar ecosystems before they are studied fully [26] and thus biodiversity data are urgently required now to determine the extent of damage that might occur.
